# Neuroprotective Effects of the Triterpenoid, CDDO Methyl Amide, a Potent Inducer of Nrf2-Mediated Transcription

**DOI:** 10.1371/journal.pone.0005757

**Published:** 2009-06-01

**Authors:** Lichuan Yang, Noel Y. Calingasan, Bobby Thomas, Rajnish K. Chaturvedi, Mahmoud Kiaei, Elizabeth J. Wille, Karen T. Liby, Charlotte Williams, Darlene Royce, Renee Risingsong, Eric S. Musiek, Jason D. Morrow, Michael Sporn, M. Flint Beal

**Affiliations:** 1 Department of Neurology and Neuroscience, Weill Medical College of Cornell University, New York-Presbyterian Hospital, New York, New York, United States of America; 2 Department of Pharmacology, Dartmouth Medical School, Hanover, New Hampshire, United States of America; 3 Department of Clinical Pharmacology, Vanderbilt University Medical Center, Nashville, Tennessee, United States of America; University of Nebraska, United States of America

## Abstract

The NF-E2-related factor-2 (Nrf2)/antioxidant response element (ARE) signaling pathway regulates phase 2 detoxification genes, including a variety of antioxidative enzymes. We tested neuroprotective effects of the synthetic triterpenoid CDDO-MA, a potent activator of the Nrf2/ARE signaling. CDDO-MA treatment of neuroblastoma SH-SY5Y cells resulted in Nrf2 upregulation and translocation from cytosol to nucleus and subsequent activation of ARE pathway genes. CDDO-MA blocked t-butylhydroperoxide-induced production of reactive oxygen species (ROS) by activation of ARE genes only in wild type, but not Nrf2 knockout mouse embryonic fibroblasts. Oral administration of CDDO-MA resulted in significant protection against MPTP-induced nigrostriatal dopaminergic neurodegeneration, pathological alpha-synuclein accumulation and oxidative damage in mice. Additionally, CDDO-MA treatment in rats produced significant rescue against striatal lesions caused by the neurotoxin 3-NP, and associated increases in the oxidative damage markers malondialdehyde, F_2_-Isoprostanes, 8-hydroxy-2-deoxyguanosine, 3-nitrotyrosine, and impaired glutathione homeostasis. Our results indicate that the CDDO-MA renders its neuroprotective effects through its potent activation of the Nrf2/ARE pathway, and suggest that triterpenoids may be beneficial for the treatment of neurodegenerative diseases like Parkinson's disease and Huntington's disease.

## Introduction

There is increasing evidence showing that both mitochondrial dysfunction and oxidative damage play a key role in the pathogenesis of neurodegenerative diseases such as Parkinson's disease (PD) and Huntington's disease (HD) [1], [2]. An extremely promising pathway for neurotherapeutics is the NF-E2-related factor-2 (Nrf2)/antioxidant response element (ARE) signaling pathway [3]–[6]. The transcription factor Nrf2 is a key regulatory factor in the coordinated induction of a battery of cytoprotective genes, including those encoding for both antioxidant and anti-inflammatory proteins. The induction of these protective genes depends on the nuclear translocation of Nrf2 and its binding to the ARE, a regulatory enhancer sequence, which is present in the promoter region of the involved genes. The regulation of the Nrf system involves another cytosolic protein, KEAP1 (kelch-like ECH-associated protein 1), which binds to Nrf2 in the cytosol and prevents its nuclear translocation and subsequent transcriptional activation of the ARE genes [7], [8]. Critical cysteine residues in the KEAP1 protein are essential for its ability to inactivate Nrf2 by retaining it in the cytosol. These cysteine residues are targets for various drugs that form Michael adducts with KEAP1 [9]–[12]. Nrf2/ARE signaling regulates over 200 genes, including those encoding anti-oxidative genes and detoxifying enzymes (known as the “phase 2” enzymes) [13].

One critical enzyme that is induced by the Nrf2 signaling pathway is NADPH quinone oxidoreductase-1 (NQO-1). This enzyme is involved in detoxification of protein-bound quinone, and it functions to maintain alpha-tocopherol and coenzyme Q_10_ (CoQ_10_) in their reduced antioxidant states. Another ARE-dependent protein is the stress responsive inducible enzyme hemeoxygenase-1 (HO-1). HO-1 is involved in the metabolism of the prooxidant heme to the antioxidant pigment biliverdin, ferrous iron and carbon monoxide [14], and it has profound anti-inflammatory properties [15]. Two inflammatory mediators which are downregulated by Nrf2 are inducible nitric oxide synthase (iNOS) and the prostaglandin synthesizing cyclooxygenase 2 (COX2) [16]. Both iNOS- and COX2-deficient mice are resistant to dopaminergic neurotoxicity in a mouse model of PD [17], [18].

Experiments using Nrf2 knockout mice and primary astroglial and neuronal cultures derived from these mice show that disruption of Nrf2 renders neuronal tissue more susceptible to oxidative stress [19]. Conversely, Nrf2 overexpression in mixed rat neuron-glia cortical cultures enhances antioxidant capacity in both neuronal and astroglial cells, and protects cortical neurons from excitotoxicity. Nrf2 deficient mice are more susceptible to 3-nitropropionic acid (3-NP) toxicity [20]. Transplantation of Nrf2 overexpressing astrocytes protects against the toxic effects of the mitochondrial complex 2 inhibitor malonate [21]. Transgenic mice over-expressing Nrf2 in astrocytes are resistant to MPTP neurotoxicity and when crossed into a transgenic mouse model of amyotrophic lateral sclerosis they delayed disease onset and prolonged survival [22], [23].

**Figure 1:**
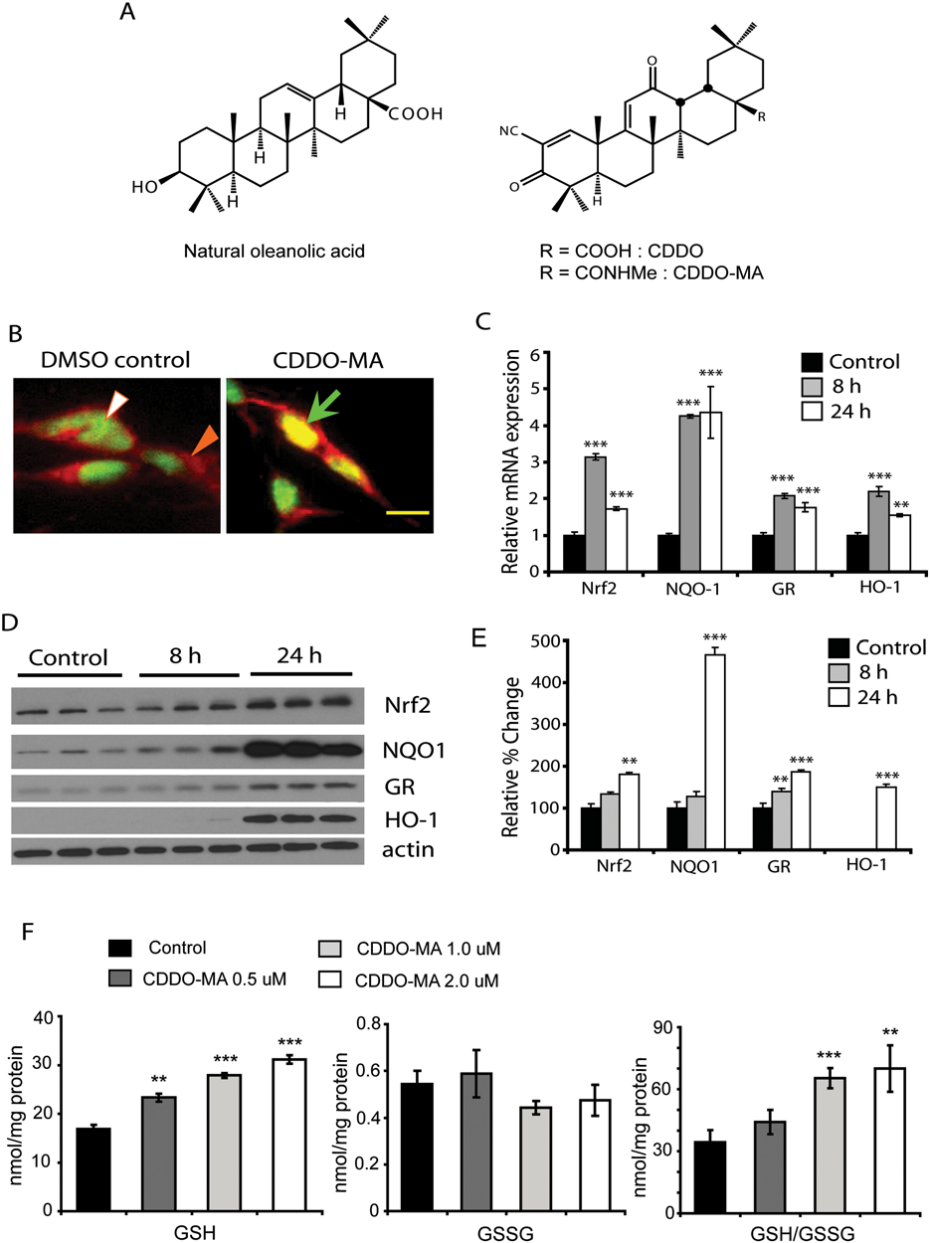
CDDO-MA treatment to SH-SY5Y cells causes Nrf2 translocation to nucleus and subsequent activation of ARE pathway. *A.* Chemical structures of oleanolic acid and CDDO-MA. Oleanolic acid is a naturally occurring triterpenoid with structural similarities to CDDO. CDDO that is over 200,000 times more potent than the parent oleanolic acid was chemically modified by addition of a methyl group to obtain increased brain bioavailability. *B.* SH-SY5Y neuroblastoma cells were treated with CDDO-MA (1 µM) or DMSO as a control. In DMSO controls Nrf2 is localized to cytosol as immunostained by anti-Nrf2 antibody (closed arrow) with DAPI (open arrow) a nuclear stain. After 8 hours of CDDO-MA treatment Nrf2 from cytosol translocates to the nucleus and colocalizes with DAPI (straight arrow), Scale bar 12 µm. *C.* Nuclear translocation of Nrf2 results in a significant increase in mRNA levels of Nrf2 and other ARE genes such as NQO-1, glutathione reductase (GR) and HO-1 measured by RT-PCR at 8 and 24 hours after drug treatment. Data represented as Mean±SEM showing relative levels of mRNA at various time points compared to DMSO controls, **p<0.05 and ***p<0.001. Students-*t* test, n = 5 samples for each experimental groups. *D and E.* Increases in mRNA levels of Nrf2 and ARE genes showed corresponding increases in levels of their respective proteins analyzed by western blotting and densitometry at 8 and 24 hours after CDDO-MA treatment. Data represented as Mean±SEM showing relative percent change in levels of protein normalized to actin at various time points compared to DMSO controls, **p<0.05 and ***p<0.001. Students-*t* test, n = 6 samples for each experimental groups. *F.* CDDO-MA treatment resulted in a significant dose-response increase in levels of reduced glutathione (GSH) measured at 24 hours. Data represented as Mean±SEM showing GSH levels at 24 hours compared to DMSO controls, **p<0.05 and ***p<0.001 following Students *t*-test (n = 6). Values represented as nanomoles/mg protein from total cell lysate.

There is, therefore, an excellent rationale for the development of new neuroprotective agents, based on their ability to enhance activity of Nrf2. Oleanolic acid is a naturally occurring triterpenoid, which has been used for centuries in Asian medicine, due to its anti-inflammatory activity. Synthetic triterpenoids such as CDDO have been found to be potent inducers of the transcriptional activity of Nrf2, resulting in marked induction of NQO-1, HO-1, glutathione transferases, and other cytoprotective enzymes, as well as suppressing induction of iNOS and COX2 at nanomolar concentrations [9], [24]–[29]. One of the possible mechanisms of these triterpenoids as Nrf2 inducers is their involvement in Michael reaction to reactive cysteine residues on KEAP1 protein [9]. In the present study, we used the synthetic triterpenoid, CDDO-methyl amide (2-cyano-*N*-methyl-3,12-dioxooleana-1,9(11)-dien-28 amide; CDDO-MA) (Fig. 1A), which is at least 200,000 times more potent than its naturally occurring distant parent, oleanolic acid, as an inducer of NQO-1 or a suppressor of iNOS [9], [25]. The cytoprotective activity of these triterpenoids is dependent on activation of Nrf2, since such activity is lost, both in cell cultures and *in vivo*, in the absence of Nrf2 [9], [26], [28]. Furthermore, pharmacodynamically meaningful levels of CDDO-MA are achievable in mouse brain after oral dosing, in contrast to its immediate carboxylic acid parent, CDDO, which has poor penetration of the blood-brain barrier.

In the present study, we found that CDDO-MA is a very potent and selective activator of the neuroprotective Nrf2/ARE pathway. Furthermore, due to its ability to activate the Nrf2 pathway, CDDO-MA exerts profound neuroprotective effects against MPTP and 3-NP neurotoxicity. We show that the neuroprotective effects observed are due to its antioxidant effects, caused by induction of pathways known to be regulated through Nrf2/ARE, such as glutathione synthesis. This is the first report to show that a synthetic oleanane triterpenoid can be neuroprotective in experimental models of PD and HD, and has potential therapeutic implications for the treatment of these disabling illnesses.

## Results

### CDDO-MA causes Nrf2 upregulation and nuclear translocation to activate the ARE pathway

Previous studies have demonstrated that synthetic triterpenoids activate Nrf2 mediated ARE transcription in non-neuronal systems [9], [26], [28]. We studied the ability of CDDO-MA to activate this pathway in a neuronal cell line. SH-SY5Y neuroblastoma cells were treated with various doses of CDDO-MA (0.5–2 µM) or DMSO as control and analyzed for cellular localization of Nrf2 at different time points. After 8 hours of CDDO-MA (1 µM) treatment Nrf2 from cytosol translocated to nucleus as evidenced by its colocalization with the nuclear dye DAPI while Nrf2 was retained in the cytosol in DMSO controls (Figure 1B). Nuclear translocation of Nrf2 from cytosol was also observed at 8 hours following CDDO-MA at doses of 0.5 and 2 µM (data not shown). Nuclear translocation of Nrf2 following CDDO-MA (1 µM) treatment resulted in significant (0.5–4.5 fold) increases in mRNA levels of Nrf2 and other ARE genes such as NQO-1, glutathione reductase (GR) and hemeoxygenase-1 (HO-1) as measured by RT-PCR at 8 and 24 hours compared to DMSO controls (Fig. 1C). Increases in mRNA levels of Nrf2 and ARE genes showed corresponding (0.5–4.8 fold) increase in levels of their proteins at 24 hours after CDDO-MA treatment determined by immunoblotting and densitometry analyses (Fig. 1D and E). Protein levels of glutathione reductase were markedly increased even at 8 hours of CDDO-MA treatment. This upregulation of glutathione reductase mRNA and its protein levels following CDDO-MA treatment resulted in a dose-dependent increase in levels of reduced glutathione (GSH), a non-significant decrease of oxidized glutathione (GSSG) and a significant increase in the GSH/GSSG ratio measured in the total cell lysate at 24 hours (Fig. 1F). The increase in GSH levels and the ratio (GSH/GSSG) were dose-dependently increased with doses of 0.5–2 µM CDDO-MA. These doses resulted in Nrf2 upregulation, its nuclear translocation, and activation of the ARE pathway (Fig. 1F and data not shown).

**Figure 2:**
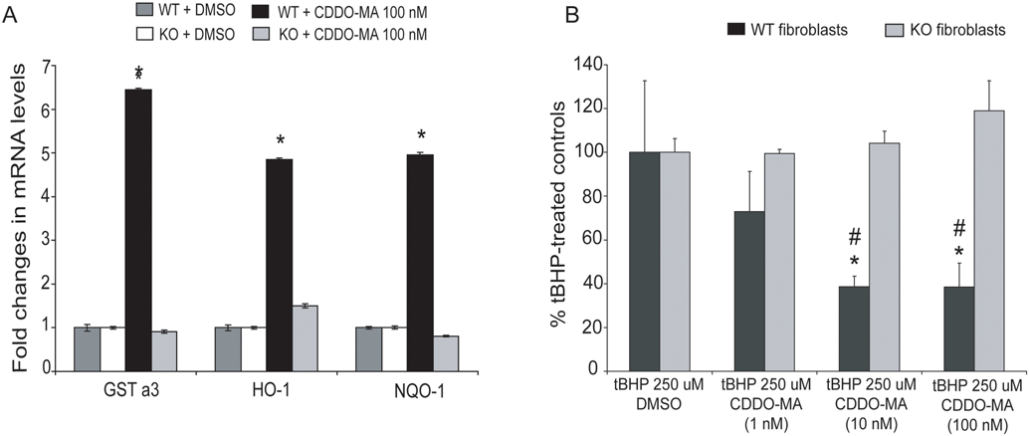
Activation of Nrf2/ARE pathway by CDDO-MA is Nrf2 dependent. *A.* Wild type (WT) and Nrf2 knockout (KO) mouse embryonic fibroblasts were pretreated with either CDDO-MA (1, 10 and 100 nM) or DMSO (as control) for 18 hours. Analysis of ARE genes showed a statistically significant increase in GSTa3, HO-1 and NQO-1 in wild type fibroblasts compared to DMSO treated controls. Nrf2 knockout fibroblasts failed to induce ARE genes following treatment with CDDO-MA compared to respective DMSO controls, implicating that CDDO-MA requires Nrf2 to activate ARE signaling. Data represent mean±SEM, *P<0.001 when compared with the controls, by ANOVA, n = 3 from two separate experiments. *B.* Wild type and Nrf2 knockout mouse embryonic fibroblasts were pretreated with either CDDO-MA (1, 10 and 100 nM) or DMSO (as control) for 18 hours. The next day, 250 µM tert-butyl hydroperoxide was added to these fibroblasts for 15 minutes, and generation of ROS was measured by flow cytometry. CDDO-MA treatment resulted in a significant dose dependent reduction of tBHP-induced ROS generation in wild type fibroblasts. Nrf2 knockout fibroblasts failed to show reduction in tBHP-induced ROS generation. *p<0.05 compared to knockout fibroblasts; #p<0.05 compared to 1 nM CDDO-MA, by ANOVA, n = 3 from two separate experiments.

### CDDO-MA activates the ARE pathway in an Nrf2-dependent manner and blocks t-butylhydroperoxide-induced ROS generation

To determine if the activation of ARE pathway by CDDO-MA is through the Nrf2-mediated transcription we studied mouse embryonic fibroblasts from wild type and Nrf2 knockout mice [30]. Following overnight treatment of CDDO-MA (100 nM) or DMSO in wild type and Nrf2 knockout fibroblasts, analysis of mRNA levels for the ARE genes such as glutathione S-transferase alpha 3 (GSTa3), HO-1 and NQO-1 were studied using RT-PCR. RT-PCR analyses of these ARE activated genes showed significant 5–6 fold inductions in wild type fibroblast cells, but not in the Nrf2 knockout cells (Fig. 2A). This suggests that indeed the ARE activation caused by CDDO-MA is through Nrf2 mediated transcription. Furthermore at a functional level we tested the ability of CDDO-MA to block t-butylhydroperoxide generated reactive oxygen species (ROS). Wild type and Nrf2 knockout mouse embryonic fibroblasts were pre-treated overnight with either CDDO-MA (1, 10, and 100 nM) or DMSO. The next day, cells were loaded with a fluorescent indicator for 30 minutes and then challenged with 250 uM t-butylhydroperoxide (a potent oxidant known to generate toxic free radicals) for 15 minutes. CDDO-MA treatment blocked t-butylhydroperoxide-induced ROS generation in wild type fibroblast cells in a dose-dependent manner, but there were no effects of CDDO-MA on t-butylhydroperoxide-induced ROS generation in Nrf2 knockout fibroblasts (Fig. 2B). CDDO-MA is, therefore, a potent and selective activator of the Nrf2/ARE pathway and can scavenge t-butylhydroperoxide-induced ROS generation through the induction of phase 2 detoxifying ARE genes only in the presence of Nrf2.

### Bioavailability of CDDO-MA in mouse brain

**Figure 3:**
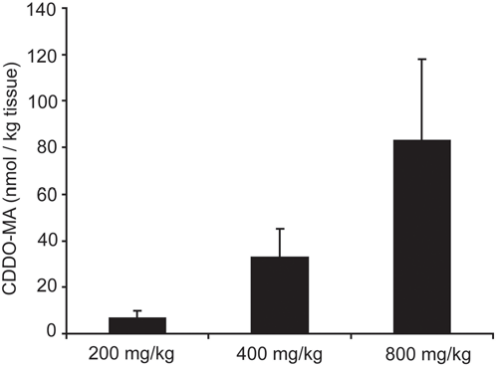
Brain bioavailability of CDDO-MA in mice. CDDO-MA shows pharmacodynamically meaningful levels in brain. A dose-dependent increase in concentration of CDDO-MA was observed using an LC-MS analysis of acetonitrile brain extracts measured following administration CDDO-MA (at 200, 400 and 800 mg/kg of rodent diet) for one week, n = 5 mice per group. Vehicle diet consisting of 1∶3 (ethanol∶Neobee oil) showed no CDDO-MA levels in brain (data not shown).

Due to its ability to activate the neuroprotective Nrf2/ARE pathway *in vitro* we then administered CDDO-MA to mice in order to test the neuroprotective efficacy of ARE activation in animal models of neurodegeneration. CDDO-MA achieves meaningful pharmacological levels in mouse brain in contrast to the naturally occurring parent oleanolic acid when administered by diet. The improved brain penetration of CDDO-MA was achieved by chemical modification of the parent oleanolic acid by replacing the carboxylic acid residue with the methyl amide group (Fig. 1A). In three separate experiments in which mice were fed CDDO-MA in the diet (800 mg/kg of diet), mass spectrometry analysis showed brain tissue levels ranging from 60–120 nM, a level associated with marked induction of phase 2 enzymes and suppression of iNOS and COX-2 in cell culture experiments [9], [25]. The dose we selected for these experiments (800 mg/kg of diet) gave brain levels that were twice those found in mice fed only 400 mg/kg diet (Fig. 3).

### CDDO-MA attenuates MPTP-induced nigrostriatal dopaminergic neurodegeneration

**Figure 4:**
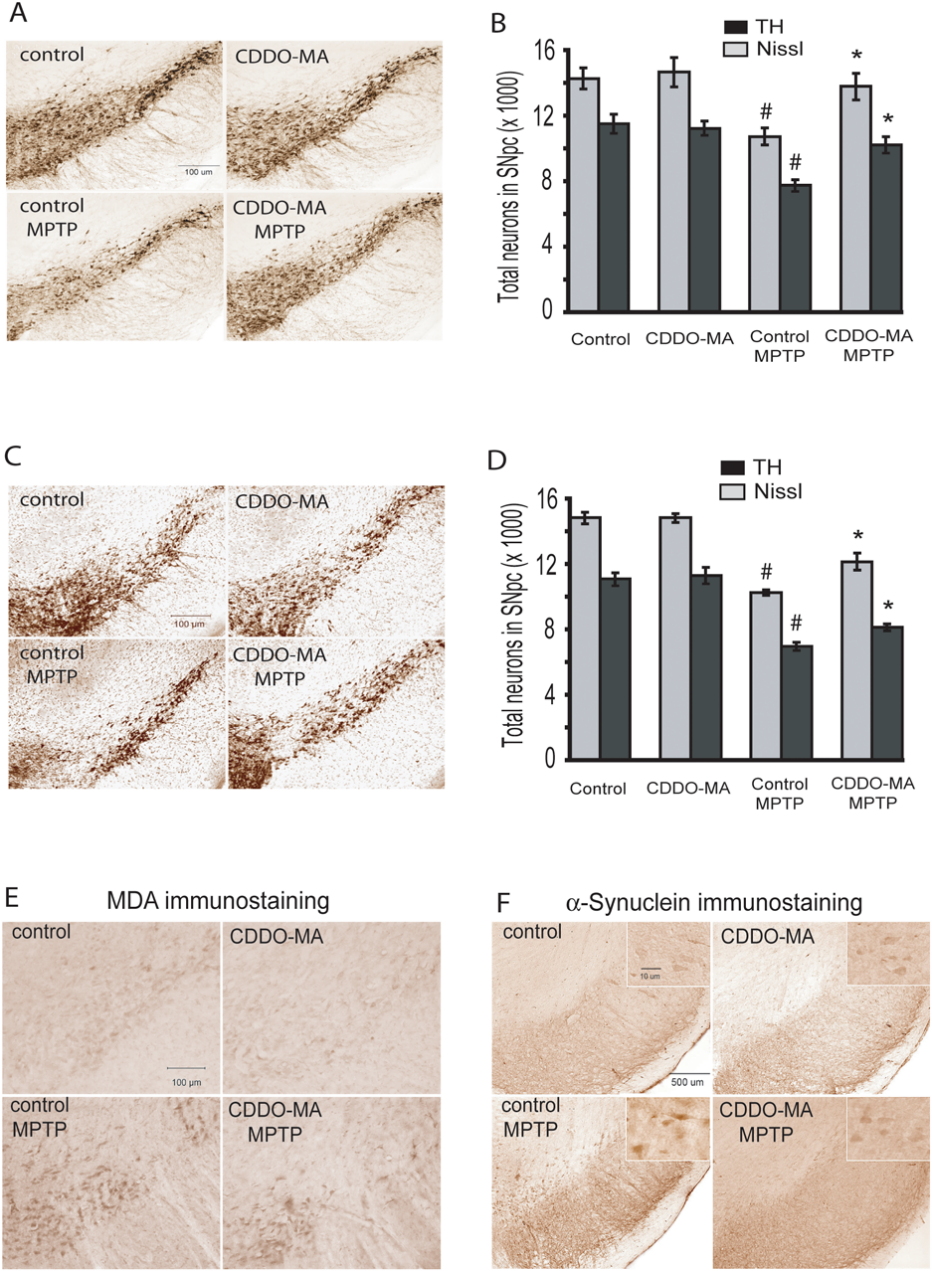
CDDO-MA attenuates MPTP-induced nigrostriatal dopaminergic neurodegeneration. Mice were pre-treated with either CDDO-MA or control diet 7 days before acute (MPTP 10 mg/kg, i.p. 3 doses every 2 hours in one day) and chronic (MPTP 40 mg/kg, for 28 day through continuous subcutaneous infusion using mini-osmotic pumps) MPTP treatment. Control animals received identical volumes of PBS. *A* Photomicrographs of TH-immunostained sections through the SNpc of mice on acute MPTP show a significant reduction in TH-positive neurons of SNpc. CDDO-MA treatment significantly blocked MPTP-induced loss of TH-positive neurons. *B.* Consistent with TH-immunostaining of SNpc, stereologic cell counts of total (Nissl-positive) and TH-immunopositive neurons of SNpc showed a significant loss following MPTP administration which was significantly attenuated by CDDO-MA treatment. *C.* Photomicrographs of TH-immunostained sections through the SNpc of mice on chronic MPTP show a significant reduction in TH-positive neurons of SNpc. CDDO-MA treatment significantly blocked MPTP-induced loss of TH-positive neurons. *D.* Consistent with TH-immunostaining of SNpc, stereologic cell counts of total (Nissl-positive) and TH-immunopositive neurons of SNpc showed a significant loss following MPTP administration which was significantly attenuated by CDDO-MA treatment. n = 10 mice per group. ^#^ *p*<0.05 compared to control diet alone; *P<0.05 when compared with the control diets with MPTP group. *E.* Photomicrographs of malondialdehyde (MDA)-immunostained sections through the SNpc of mice on chronic MPTP show a significant increase of MDA staining in SNpc of MPTP treated animals. CDDO-MA treatment resulted in a marked reduction of MPTP-induced MDA formation. Representative image from n = 5 mice in each group. *F.* Photomicrographs of α-synuclein stained sections through the SNpc of mice on chronic MPTP show a significant increase of α-synuclein accumulation in SNpc of MPTP treated animals. CDDO-MA treatment resulted in a significant reduction of MPTP-induced α-synuclein accumulation in SNpc neurons. High magnification inserts show the α-synuclein staining in neurons. Representative image from n = 5 mice in each group.

We examined whether the ability of CDDO-MA to activate Nrf2/ARE signaling is beneficial in an experimental animal model of Parkinson's disease. Mice were administered either CDDO-MA (800 mg/kg of diet) or control rodent diet for 7 days before MPTP (10 mg/kg, i.p. 3 doses every 2 hours in one day) and then for 7 days after MPTP. One week following the last MPTP treatment, there was a profound reduction (about 80%) in total striatal dopamine and also its metabolites 3,4-dihydroxyphenylacetic acid (DOPAC), homovanillic acid (HVA) as compared to phosphate-buffered saline (PBS) controls. CDDO-MA treatment significantly attenuated MPTP-induced striatal depletion of dopamine and its metabolites as compared to the MPTP group on control diet (Table 1) (ANOVA, p<0.01). Immunohistochemical analysis of dopaminergic neurons of the substantia nigra pars compacta (SNpc) showed a profound loss of tyrosine hydroxylase (TH)-immunoreactivity 7 days after acute MPTP as compared to PBS controls. CDDO-MA treatment significantly protected against the loss of TH-immunoreactive dopaminergic neurons in the SNpc (Fig. 4A). Counts of total neurons (Nissl positive) and TH-immunopositive neurons in the SNpc showed that treatment with CDDO-MA significantly reduced the MPTP-induced loss of both total and TH-immunopositive neurons (Fig. 4B) (ANOVA, p<0.05).

**Table 1:** Neuroprotective effects of CDDO-MA on striatal dopamine and its metabolites in MPTP neurotoxicity.

	Animal (n)	Dopamine	DOPAC	HVA
*Acute MPTP model*				
Control+PBS	5	90.10±1.7	7.04±0.2	12.47±0.6
CDDO-MA+PBS	5	85.35±3.6	7.06±0.3	11.58±0.7
Control+MPTP	10	17.86±1.1##	2.17±0.1##	5.20±0.1##
CDDO-MA+MPTP	10	46.22±6.3## **	4.28±0.4## **	8.26±0.5## **
*Chronic MPTP model*				
Control+PBS	5	87.73±2.6	8.00±0.2	9.78±0.4
CDDO-MA+PBS	5	86.99±3.5	7.88±0.3	9.20±0.3
Control+MPTP	13	41.60±4.6##	1.57±0.1##	4.05±0.2##
CDDO-MA+MPTP	15	60.07±4.7# *	2.32±0.3# *	5.10±0.2# *

a Data are expressed as mean±SEM. Values represent as ng per mg protein.

b #, p<0.05, ##, p<0.01compared to PBS groups; *, p<0.05, **, p<0.01 compared to control diet +MPTP group, ANOVA, Student-Newman-Keul's post test.

We then studied CDDO-MA in a chronic paradigm of MPTP-toxicity. The chronic MPTP model better mimics the neuropathological features of PD in humans [31], [32]. Mice were pre-treated with either CDDO-MA or control diet. Seven days later animals received a continuous infusion of MPTP (40 mg/kg/day for 28 days) or sodium phosphate-buffered saline (PBS) by subcutaneous implantation of mini-osmotic pumps. Animals were administered either CDDO-MA or control diet through out the duration of the osmotic pumps. After 28 days, there was a significant reduction (about 50%) in total striatal dopamine and also its metabolites, DOPAC and HVA. CDDO-MA treatment significantly protected against MPTP-induced striatal depletion of dopamine and its metabolites (Table 1) (ANOVA p<0.05). Immunohistochemical analysis of the SNpc after 28 days showed a marked reduction of total (Nissl positive) and TH-immunoreactive neurons following MPTP. CDDO-MA treatment significantly protected against MPTP-induced loss of TH-immunoreactive and total neurons of the SNpc (Fig. 4C and D) (ANOVA, p<0.05).

The neuroprotective effects provided by CDDO-MA treatment following MPTP-neurotoxicity were not due to impairment of the metabolism of MPTP, since the striatal tissue levels of its metabolite MPP^+^ (1-methyl-4-phenyl-pyridinium ion) showed no differences between the control (9.85±0.69 ng/mg protein, n = 13) and the CDDO-MA treated mice (9.83±0.50 ng/mg protein, n = 15), when measured after 28 days of chronic treatment of MPTP (40 mg/kg/day for 28 days).

### CDDO-MA blocks MPTP-induced lipid peroxidation and α-synuclein accumulation

In order to determine if neuroprotective effects of CDDO-MA can block MPTP-induced oxidative damage and increase in α-synuclein accumulation we examined malondialdehyde and α-synuclein immunostaining in SNpc. Chronic administration of MPTP resulted in a marked induction of oxidative damage as measured by malondialdehyde immunostaining (Fig. 4E), and caused a marked increase in α-synuclein accumulation in SNpc dopaminergic neurons (Fig. 4F). MPTP-induced α-synuclein accumulation was observed in TH-positive neurons of SNpc as determined by colocalization of α-synuclein- and TH-immunostaining employing confocal microscopy (data not shown). Treatment with CDDO-MA blocked MPTP-induced mesencephalic malondialdehyde formation and accumulation of α-synuclein (Fig. 4E and F). These data show that CDDO-MA produces neuroprotective effects by reducing oxidative stress. It also reduces α-synuclein accumulation in the SNpc.

### CDDO-MA blocks 3-nitropropionic acid-induced striatal toxicity

**Figure 5:**
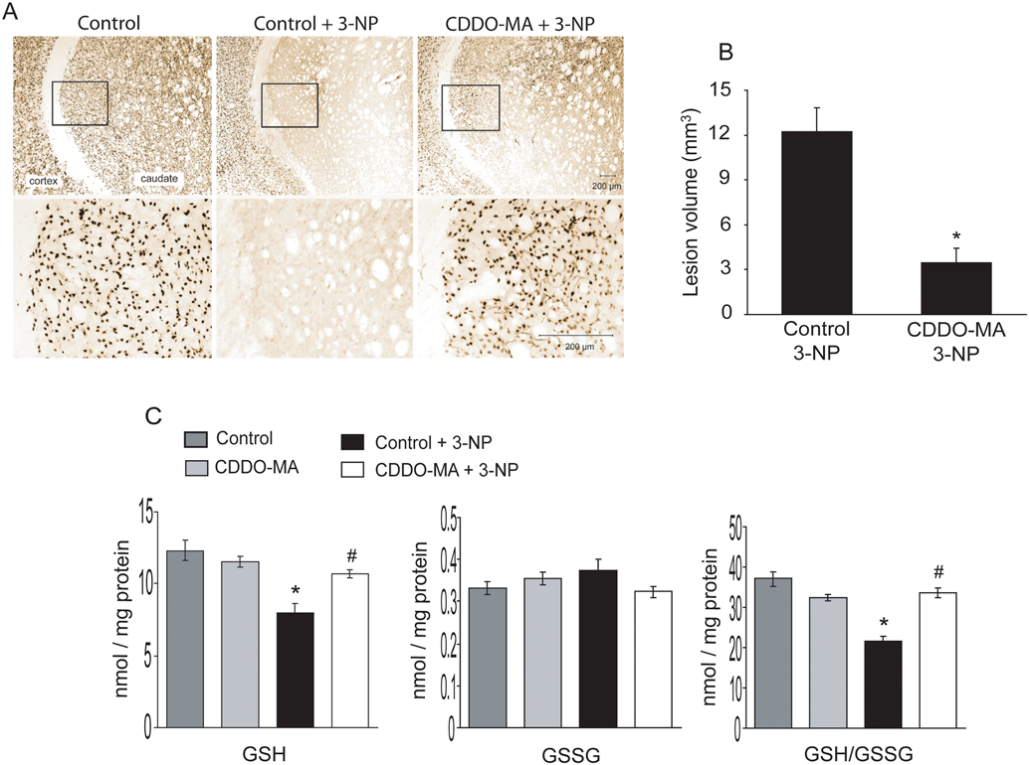
CDDO-MA reduces striatal damage caused by 3-NP toxicity, and blocks 3-NP induced striatal glutathione depletion. Lewis rats were pre-treated with either CDDO-MA diet (800 mg/kg) or control diet 7 days followed by continuous infusion of 3-NP (50 mg/kg/day) delivered through subcutaneously implanted miniosmotic pumps for 7 days. *A.* Photomicrographs of NeuN-immunostained sections through the coronal section of striatum and cortex of rats treated with control diet+PBS (left), control diet with 3-NP (middle) and CDDO-MA diet with 3-NP (right) show a significant loss of NeuN positive cells in 3-NP treated cortex and caudate compared to PBS controls. CDDO-MA treatment significantly reduced 3-NP induced loss of cortical and caudate NeuN positive cells. High magnifications in lower panel show the striatal neuronal loss caused by 3-NP and preservation by CDDO-MA. *B.* Consistent with loss of NeuN positive cells in cortex and the caudate, measurement of lesion volume in coronal brain slices of 3-NP treated controls show a significant increase in striatal lesion volumes. The administration of CDDO-MA significantly reduces striatal lesion volumes caused by 3-NP. Data represent mean±SEM. *p<0.01, statistical significance compared to 3-NP treated controls, by student-*t* test, n = 15 rats per group. *C.* Rats described above were also used for analysis of striatal levels of reduced and oxidized glutathione. 3-NP administration resulted in a statistically significant reduction of striatal levels of reduced glutathione (GSH) and the ratio of reduced and oxidized glutathione (GSH/GSSG), whereas levels of oxidized glutathione (GSSG) were not significantly affected by 3-NP treatment measured by HPLC-electrochemistry. CDDO-MA treatment significantly blocked 3-NP induced reductions in striatal levels of GSH and the ratio of GSH/GSSG without impacting the GSSG levels. Data represent mean±SEM. *p<0.05, statistical significance compared to controls and ^#^ *p*<0.05 compared to 3-NP treatment, by ANOVA, n = 10 rats per group.

3-Nitropropionic acid (3-NP) administration in rodents is known to cause striatal degeneration known to replicate pathological features of Huntington's disease [33]. Lewis rats were pretreated either with CDDO-MA (800 mg/kg of diet) or control diet for one week. 3-NP (50 mg/kg/day for 7 days) or PBS was delivered chronically by subcutaneous implantation of mini-osmotic pumps. Animals were treated with either CDDO-MA or control diet throughout the administration of 3-NP. Analysis of striatal neurons by neuron-specific nuclear protein (NeuN) immunohistochemistry after 3-NP administration showed a profound loss of striatal neurons. CDDO-MA treatment significantly attenuated 3-NP-induced loss of striatal NeuN stained neurons (Fig 5A). Quantitative assessment showed that 3-NP induced striatal lesion volumes were reduced by more than 70% by administration of CDDO-MA (Fig. 5B) (p<0.01).

### CDDO-MA preserves 3-NP-induced impairment of striatal glutathione homeostasis, and blocks oxidative damages

Striatal degeneration caused by 3-NP administration in rodents induces oxidative damage, especially alterations in glutathione homeostasis [34]. We pretreated rats either with CDDO-MA (800 mg/kg of diet) or control diet for one week followed by mini-osmotic pump delivery of 3-NP (50 mg/kg/day for 7 days). Administration of 3-NP resulted in a significant reduction of reduced glutathione (GSH), and the ratio of reduced to oxidized glutathione (GSH/GSSG). Treatment with CDDO-MA significantly blocked 3-NP-induced depletion of striatal GSH and the ratio of GSH/GSSG (Fig. 5C).

**Figure 6:**
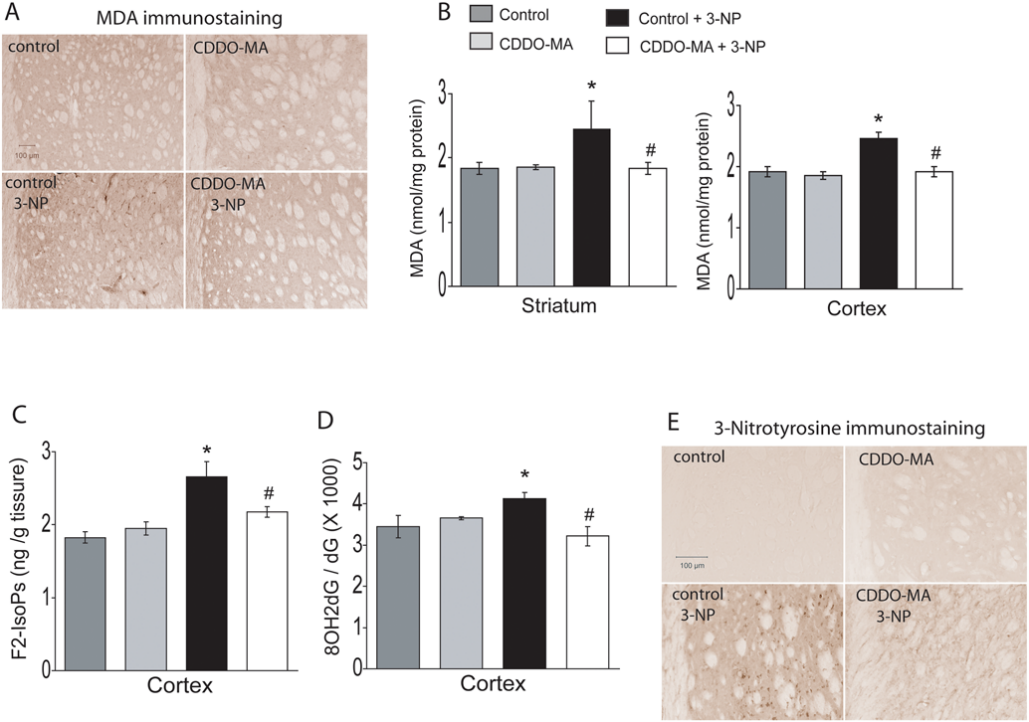
CDDO-MA treatment blocks 3-NP induced oxidative damage. Lewis rats described in figure 4 were also used for striatal and cortical levels of oxidative damage markers. *A.* Photomicrographs of malondialdehyde (MDA)-immunostained coronal sections of the striatum of rats administered with 3-NP shows an increase in MDA staining in striatum. Treatment with CDDO-MA markedly reduced 3-NP induced MDA immunostaining in the striatum, n = 5 mice per group, scale bar: 100 µm *B.* Consistent with the striatal immunostaining of MDA, HPLC-electrochemical analysis of MDA levels in striatum and cortex show 3-NP resulted in a statistically significant increase in MDA levels. CDDO-MA treatment significantly reduced 3-NP induced MDA formation in striatum and cortex. Data represent mean±SEM. *p<0.05 compared to normal controls and ^#^ *p*<0.05 compared to 3-NP treatment, by ANOVA, n = 10 rats per group. *C.* Gas chromatography/Mass spectrometric analysis of esterified F_2_-Isoprostanes (F_2_-IsoPs) in cerebral cortex from rats treated with 3-NP showed significant increases in F_2_-IsoPs levels. CDDO-MA treatment significantly abrogated 3-NP induced cortical increases in F_2_-IsoPs levels. Data represent mean±SEM. *p<0.05 compared to normal controls and ^#^ *p*<0.05 compared to 3-NP treatment, by ANOVA, n = 10 rats per group. *D.* HPLC-electrochemical analysis of the ratio of 8OH2dG over dG shows a significant increase in oxidative DNA damage in the cerebral cortex following 3-NP treatment which is significantly attenuated due to CDDO-MA administration. Data represent mean±SEM. *p<0.05 compared to normal controls and ^#^ *p*<0.05 compared to 3-NP treatment, by ANOVA, n = 10 rats per group. *E.* Photomicrographs of 3-nitrotyrosine-immunostained coronal brain sections through the striatum of rats show a significant elevation in 3-nitrotyrosine levels (a marker of protein nitration) due to 3-NP administration. Treatment of CDDO-MA significantly reduced 3-NP induced increase in immunostaining of striatal 3-nitrotyrosine, n = 5 rats per group, scale bar: 100 µm.

Immunohistochemical analysis of striatal slices after 3-NP showed a marked increase in malondialdehyde staining. CDDO-MA treatment completely blocked 3-NP induced malondialdehyde staining in the striatum (Fig. 6A). Quantitative measurement of malondialdehyde by high-performance liquid chromatography (HPLC) revealed a significant increase of malondialdehyde both in striatum and cerebral cortex following 3-NP treatment. 3-NP induced elevations of malondialdehyde levels in these two areas were significantly attenuated by CDDO-MA treatment (Fig. 6B). F_2_-Isoprostanes, a marker of lipid peroxidation, showed a marked increase after 3-NP treatment. CDDO-MA significantly blocked 3-NP induced generation of F_2_-Isoprostanes (Fig. 6C). Furthermore, administration of 3-NP resulted in DNA oxidation as measured by the ratio of 8-hydroxy-2-deoxyguanosine (8OH2dG)/deoxyguanosine (dG) in the cerebral cortex, which was significantly attenuated by CDDO-MA administration (Fig. 6D). 3-NP administration produced a profound increase in striatal immunostaining of 3-nitrotyrosine, a nitrated tyrosine marker for protein oxidation, which was markedly attenuated by treatment with CDDO-MA (Fig. 6E). These results suggest that CDDO-MA blocks oxidative damage seen in 3-NP model in rats.

Overall, our results show that CDDO-MA is a selective and potent activator of the Nrf2/ARE signaling pathway and activation of this pathway provides neuroprotective effects in experimental models of PD and HD.

## Discussion

A promising pathway for neurotherapeutics in neurodegenerative diseases is the Nrf2/ARE signaling pathway [3]–[5]. The ability to induce neuroprotective phase 2 detoxifying genes, as well as antioxidant enzymes at transcriptional level, could have significant advantages over more conventional approaches. One can potentially induce an ongoing neuroprotective response which would not be dependent on the antioxidant effects of single molecules, which are frequently consumed while scavenging reactive oxygen species (ROS).

Under basal conditions, Nrf2 is anchored in the cytoplasm to the actin bound protein KEAP1. In response to oxidative stress Nrf2 is released from the KEAP1/Nrf2 complex and then activates ARE-mediated gene transcription [7], [8], [10]. Genes known to have ARE response elements include glutathione-S-transferases, NQO-1, HO-1, thioredoxin, and enzymes involved in glutathione synthesis. A number of other genes are also under the regulatory control of Nrf2/ARE signaling which are involved in immune function and inflammation [3]. Furthermore, a number of growth factors including nerve growth factor, fibroblast growth factor, and brain derived neurotrophic factor are induced in primary neurons by activation of the Nrf2/ARE pathway [3], [13]. Additionally, a number of enzymes involved in glycolytic metabolism, as well as heat shock proteins, and ferritin light and heavy chains are also induced. Of particular interest are iNOS and COX-2 which are downregulated due to activation of Nrf2 signaling. These pro-inflammatory genes have been implicated in inflammatory pathways involved in a number of neurodegenerative diseases including PD, Alzheimer's disease and amyotrophic lateral sclerosis [17], [35]–[38].

Thus pharmacological agents that have the ability to activate the Nrf2/ARE signaling pathway hold great promise for therapeutic intervention in neurodegenerative diseases. One such class of compounds known to activate this pathway is the triterpenoids [12]. Triterpenoids are synthesized by the cyclization of squalene, a triterpene hydrocarbon found ubiquitously in nature including plants and humans. More than 20,000 triterpenoids are known to occur in nature of which oleanolic acid has been used for centuries in traditional Asian medicine, due to its anti-inflammatory activity. Using an assay for iNOS, a derivative of oleanolic acid CDDO (2-cyano-3, 12-dioxooleana-1,9-dien-28-oic acid) was identified that is over 200,000 times more active than its parent molecule oleanolic acid [12]. Structure-activity analysis shows that α, β-unsaturated carbonyl groups in CDDO at key positions on rings A and C are essential for enabling Michael addition with a nucleophilic target similar to Keap 1 (a protein with multiple nucleophilic –SH residues and a cytoplasmic repressor of Nrf2). This reaction activates the phase 2 responses, an intrinsic mechanism used by cells to deactivate electrophiles or oxidative stress [12]. In the present study, human neuroblastoma cells treated by CDDO-MA demonstrated a stream of changes in the phase 2 response through Nrf2/ARE pathway activation. These include induction of Nrf2 protein levels and subsequent nuclear translocation from the cytosol. The nuclear translocation of Nrf2 resulted in induction of phase 2 genes encoding a coordinated family of cytoprotective proteins that include the enzymes of glutathione synthesis and transfer, the quinone oxidoreductase NQO-1, and the hemeoxygenase HO-1. Significantly, the phase 2 response in neuroblastoma cells due to CDDO-MA was translated functionally by a dose dependent increase in cellular GSH production, one of the major cellular antioxidants under the regulatory control of Nrf2/ARE signaling [20]. Another significant finding in our study is that the phase 2 response due to Nrf2/ARE activation by CDDO-MA is selective and depends on the presence of a functional Nrf2. In mouse embryonic fibroblast cells derived from wild type mice CDDO-MA treatment resulted in induction of mRNA levels for ARE genes such as GSTa3, NQO-1 and HO-1. The induction of these ARE genes was completely absent in embryonic fibroblasts cells from Nrf2 knockout mice. This effect was consistent with the dose-dependent reduction of t-butylhydroperoxide-induced ROS in wild type but not Nrf2 knockout fibroblast cells. It is thus plausible that CDDO-MA may very well enable Michael's addition to its target Keap1 which is the cytosolic repressor of Nrf2, since induction of ARE genes require presence of functional Nrf2 and its translocation from cytosol to nucleus. Previously, the ability of synthetic triterpenoids to activate the ARE pathway in other model systems was demonstrated [9], [26], [28]. However, our results in the present study for the first time have shown that synthetic triterpenoids such as CDDO-MA are potent inducers of Nrf2 expression, and activators of the ARE pathway in a neuronal cell line.

Previous *in vitro* studies showed that neuronal cultures derived from Nrf2 knockout mice have increased susceptibility to oxidative stress, as well as damage produced by mitochondrial electron transport chain complex inhibitors such as rotenone, MPP^+^, 3-NP and malonate [19]. Furthermore, Nrf2 deficient mice show profound susceptibility to striatal lesions produced by the mitochondrial complex 2 inhibitor malonate [21]. Conversely, the implantation of intrastriatal grafts of astrocytes overexpressing Nrf2, produced neuroprotection against the neurotoxic effects of malonate. Recently, transgenic mice overexpressing Nrf2 in astrocytes were shown to be resistant to MPTP toxicity and to extend survival when crossed with a transgenic mouse mode of ALS [22], [23]. In order to study the neuroprotective efficacy of CDDO-MA induced activation of Nrf2/ARE pathway *in vivo* we used two neurotoxic paradigms of MPTP, involving acute 7-day and chronic continuous infusion for four weeks in mice. Administration of CDDO-MA produced significant neuroprotection against loss of striatal dopamine and its metabolites. Furthermore, it produced significant protection against loss of tyrosine hydroxylase immunoreactive neurons in the SNpc. These data are consistent with MPTP-induced enhanced susceptibility to nigrostriatal dopaminergic neurodegeneration in Nrf2 knockout mice, suggesting that impairment of Nrf2/ARE signaling is an important mediator of dopaminergic neurodegeneration [39]. Additionally, in the chronic MPTP neurotoxicity paradigm, there was increased accumulation of α-synuclein (a neuropathological feature seen in PD) in dopaminergic neurons, which was blocked by administration of CDDO-MA. This is consistent with other reports where chronic administration of MPTP, and other toxins which specifically target mitochondria such as rotenone and paraquat, produce mitochondrial dysfunction leading to oxidative stress resulting in α-synuclein accumulation in SNpc neurons [31], [32], [40], [41]. Interestingly, mice lacking α-synuclein are resistant to mitochondrial toxins [31], [42] whereas human A53T α-synuclein-overexpressing mice are known to develop neuronal mitochondrial degeneration and cell death through induction of oxidative stress [43], [44]. This notion gains support from a recent study where activation of phase 2 detoxification enzymes blocked oxidative stress induced dopaminergic neurodegeneration in a α-synuclein fly model [45]. Notably, in our studies chronic MPTP-induced oxidative damage in SNpc neurons as measured by malondialdehyde staining was markedly blocked by CDDO-MA administration. This effect of CDDO-MA is consistent with its ability to activate ARE genes in fibroblasts to scavenge t-butylhydroperoxide induced ROS generation.

Previous reports have demonstrated that Nrf2 knockout mice are more sensitive to 3-NP toxicity [20]. In cultured astrocytes, 3-NP increases Nrf2 activity, which may account for the increased sensitivity of Nrf2 deficient animals to 3-NP toxicity *in vivo* [20]. Additionally, dietary supplementation with the Nrf2 inducer tertbutylhydroquinone attenuated 3-NP toxicity in Nrf2 competent mice, but not in Nrf2 deficient mice [20]. To further demonstrate that activation of Nrf2/ARE pathway by CDDO-MA could be beneficial in a rodent model of 3-NP mediated HD, we employed systemic administration of 3-NP toxicity in Lewis rats [46]. Our findings demonstrate that administration of CDDO-MA reduced the striatal lesion volumes by more than 70%. Histological evaluation confirmed that CDDO-MA significantly protects against 3-NP induced neuronal death. Additionally, we observed that CDDO-MA blocked 3-NP induced increases in 8-hydroxy-2-deoxyguanosine, a marker of oxidative damage to DNA, and malondialdehyde levels in the striatum and cortex. Increases in F_2_-Isoprostanes (F_2_-IsoPs) in cerebral cortex produced by 3-NP were blocked by CDDO-MA. F_2_-Isoprostanes are prostaglandin-like lipids derived from the free-radical mediated peroxidation of arachidonic acid [47], [48]. The measurement of F_2_-IsoPs by GC/MS is considered the most accurate index of lipid peroxidation *in vivo*, and elevated levels of tissue-esterified and free F_2_-IsoPs have been described in numerous neurodegenerative diseases [48], including Huntington's disease [49].

Increases in 3-NP induced immunostaining for malondialdehyde and 3-nitrotyrosine, a marker of protein nitration, were blocked by CDDO-MA administration. Furthermore, increased production of glutathione, due to induction of glutathione synthetic enzymes is a well known effect of increased expression of the Nrf2/ARE genes. We found that administration of 3-NP significantly reduced striatal concentrations of reduced glutathione (GSH), and the reduction in GSH and the ratio of GSH/GSSG were completely prevented by CDDO-MA administration. This is consistent with adenoviral overexpression of Nrf2 in the striatum, which mainly infects astrocytes, which was sufficient to reduce 3-NP toxicity *in vivo* by synthesis of GSH [20]. This view is further strengthened when only a small number of Nrf2 expressing astrocytes can confer neuroprotection to many neighboring neurons during an oxidative glutamate insult *in vitro* [3], [21], [50]. These Nrf2 overexpressing astrocytes secrete high levels of glutathione, providing a means for a small number of astrocytes to affect the survival of a much larger number of neurons. Similar observations were made in transgenic mice overexpressing Nrf2 in astrocytes. When these mice were crossed with two transgenic mouse models of familial amyotrophic lateral sclerosis, they delayed onset and extended survival [23]. Motor neuron protection was dependent on glutathione secretion *in vitro* and spinal cord glutathione levels were increased *in vivo*. Our results demonstrating a dose-dependent increase of cellular GSH levels in neuroblastoma cells following CDDO-MA treatment, also suggests that CDDO-MA not only impacts glutathione synthesis in astrocytes *in vivo,* but also may activate inherent neuronal GSH synthesis to provide neuroprotection against 3-NP neurotoxicity.

In summary the present study demonstrates that CDDO-MA is a potent activator of Nrf2/ARE signaling pathway and renders neuroprotection against MPTP and 3-NP neurotoxicity. We recently showed that CDDO-MA reduces β-amyloid deposition and improves memory in a transgenic mouse model of Alzheimer's disease [51]. In that study we showed that administration of CDDO-MA increased brain levels of HO-1, an Nrf2/ARE dependent enzyme. These results provide compelling evidence for the utility of pharmacological agents known to activate Nrf2/ARE mediated phase 2 detoxification genes for the treatment of neurodegenerative diseases. Currently the parent compound, CDDO, and its close relative, CDDO-methyl ester, are in Phase I studies for the treatment of cancer. For applications in neurodegenerative disease, the superior pharmacokinetics of CDDO-MA in the brain provides a rationale for further investigation of this agent, as well as related triterpenoid amides. Our data provide a strong rationale for further preclinical investigation of CDDO-MA and other related amides, directed toward eventual clinical trials in patients with Parkinson's and Huntington's disease.

## Materials and Methods

### Cell Culture studies

Human neuroblastoma SH-SY5Y cells (ATCC, Manassas, VA) were cultured in DMEM supplemented with 10% FBS, 100 U/ml penicillin, and 100 µg/ml streptomycin at 37°C in a humidified atmosphere of 5% CO_2_. Cells were cultured up to 80–90% confluency in 10 cm dishes for real time PCR, western blotting and measurement of GSH and GSSG. For western blot analysis cells were harvested in lysis buffer (50 mm Tris–HCl, pH 7.4, 150 mm NaCl, 5 mm EDTA, 1% sodium dodecyl sulfate (SDS)) supplemented with the protease inhibitor cocktail (EDTA-free, Roche) and a phosphatase inhibitor cocktail (Sigma). Ten µg of the total protein were subjected to immunoblot analyses by a described method [32] using appropriate primary antibodies (anti-Nrf2, (1∶1000 Epitomics CA); anti-NQO1, anti-glutathione reductase and anti-heme oxygenase-1 (1∶1000 Abcam MA) and anti-β-actin Chemicon 1∶10,000)). Densitometric analyses were performed using the NIH Image J Software.

Nrf2 immunostaining was performed in SH-SY5Y cells cultured at a density of 1×10^−6^ cells/ml in 4 well chamber slides (BD Biosciences CA). Cells were fixed at room temperature with 2% paraformaldehyde in phosphate buffered saline (PBS), washed three times in PBS for 5 min, and permeabilized with 0.1% TritonX-100 in PBS. Non-specific antibody binding was blocked with 5% normal goat serum (NGS) in PBS for 1 h at room temperature. Then, cells were incubated overnight at 4°C with rabbit monoclonal anti-Nrf2 antibody (1∶200 in PBS plus 1.5% NGS, Epitomics CA), washed three times with PBS, and further incubated with Cy3-conjugated anti-rabbit IgG (1∶500 in PBS plus 0.1% NGS, Jackson Immunoresearch, PA) for 2 h at room temperature. They were washed in PBS three times for 5 minutes each at room temperature and incubated with 4′,6-diamidino-2-phenylindole, dilactate (DAPI, dilactate) (1∶1000, Invitrogen, Carlsbad, CA) for 10 minutes and rinsed in distilled water, air dried and cover slipped using immumount mounting media (Thermo Shandon, Pittsburg, PA). For microscopic analysis, the sections were visualized using a Zeiss Confocal microscope (LSM 510 Meta) (Carl Zeiss Vision, Hallbergmoos, Germany) equipped with an argon laser. DAPI immunofluorescence was given a pseudo green color in order to demonstrate a clear contrast for Nrf2 co-localization with nuclear stain DAPI following CDDO-MA treatment.

### RNA isolation and real-time RT-PCR

Total RNA from SH-SY5Y and mouse embryonic fibroblast cells were isolated according to manufacturer's protocol using TRIzol reagent (Invitrogen CA). Total RNA purity and integrity was confirmed by ND-1000 NanoDrop (NanoDrop Technologies) and 2100 Bioanalyzer (Agilent). Real-time PCR was performed using the ABI prism 7900 HT sequence detection system (Applied Biosystems, Foster City, CA) based on the 5′-nuclease assay [52] for various genes indicated and housekeeping gene GAPDH. Relative expression was calculated using the ΔΔ Ct method [53].

### Measurement of reactive oxygen species in mouse embryonic fibroblasts

Wild type and Nrf2 knockout mouse embryonic fibroblasts were treated with CDDO-MA for 18 hours and incubated with 10 µmol/L nonfluorescent indicator H_2_DCFDA (2′, 7′-Dichlorodihydrofluorescein diacetate, Invitrogen CA) for 30 minutes. Cells were challenged with 250 µmol/L tert-butyl hydroperoxide (tBHP) for 15 minutes, and mean fluorescence intensity of 10,000 cells was analyzed by FACScan flow cytometry (Becton Dickinson, NJ) using a 480-nm excitation wavelength and a 525-nm emission wavelength.

### Animals and materials

Male C57BL/6 mice (3-month old, 25–30 g) and male Lewis rats (3-month old, 250–300 g) were obtained from the Jackson Laboratory (Bar Harbor, ME, USA). Neurotoxins MPTP and 3NP, neurochemicals dopamine, DOPAC and HVA, and malondialdehyde (MDA) were all purchased from Sigma (St. Louis, MO, USA). CDDO-MA was synthesized by the condensation of methyl amide with CDDO acid chloride [25]. This stable derivative was blended into powdered rodent chow (Lab Diets 5002), after being dissolved in a vehicle comprising of 1 part ethanol/3 parts Neobee Oil (coconut oil triglyceride). For each kg of diet, 800 mg of CDDO-MA was dissolved in 50 ml of the above vehicle before mixing with the chow; the resulting diet was then pelleted for feeding. CDDO-MA is stable (by mass spec analysis) in this feed. All animal experimental procedures were in strict accordance with protocols approved by the Weill Cornell Medical College Institutional Animal Care and Use Committee (IACUC). MPTP and 3-NP manipulations were carried out in a special animal room restricted for neurotoxin manipulation.

### MPTP neurotoxicity paradigms and CDDO-MA treatments

C57BL/6 mice were fed with CDDO-MA-containing or control diet for one week before MPTP injection [54]. MPTP-HCl dissolved in PBS was injected intraperitoneally 10 mg/kg, free base for 3 times with an interval of 2 hours. Diets were continued for 7 days after the MPTP injection till they were sacrificed for toxicity analyses. Another group of C57BL/6 mice fed with CDDO-MA-containing or control diet for one week were implanted subcutaneously with osmotic minipumps (Model 2004, Alzet, Cupertino CA, filled with MPTP 170 mg/ml in PBS) that delivered MPTP at a dose of 40 mg/kg body weight daily for 28 day. They were maintained on the same diets. Then mice were sacrificed and tissues were prepared as above except that a piece of striatum from each mouse was saved for MPP^+^ level measurements.

### Rat model of 3-NP toxicity

Male Lewis rats fed with CDDO-MA-containing or control diet for 7 days were implanted subcutaneously with osmotic minipumps (Model 2ML1, Alzet, Cupertino CA, filled with 3-NP 54 mg/ml in PBS, pH 7.4) that delivered 3-NP 50 mg per kg bodyweight daily for 7 days while remaining on the same diets [46]. Then rats were sacrificed and one half of the brain was fixed in 4% paraformaldehyde for the striatal lesion volume measurement and the other half was dissected freshly for GSH, GSSG, F_2_-Isoprostanes (F_2_-IsoPs) and MDA assays.

### HPLC analysis for catecholamines and MPP^+^

Striatal levels of dopamine and its metabolites DOPAC and HVA were measured after sonication and centrifugation in chilled 0.1 M perchloric acid (PCA, about 100 ul/mg tissue) as modified from our previously described method [55]. Briefly, 15 ul supernatant was isocratically eluted through an 80×4.6 mm C18 column (ESA, Inc Chelmsford, MA) with a mobile phase containing 0.1 M LiH_2_PO_4_, 0.85 mM 1-octanesulfonic acid and 10% (v/v) methanol and detected by a 2-channel Coulochem II electrochemical detector (ESA, Inc. Chelmsford, MA). Concentrations of dopamine, DOPAC and HVA are expressed as ng per mg protein. The protein concentrations of tissue homogenates were measured according to the Bio-Rad protein analysis protocol (Bio-Rad Laboratories, Hercules, CA) and Perkin Elmer Bio Assay Reader (Norwalk, CT). MPP^+^ was measured according to described method [56].

### HPLC analysis for malondialdehyde

The determination of malondialdehyde by HPLC was carried out according to a reported method [57]. Briefly, tissues were homogenized in 40% ethanol solution. To a 50-µl aliquot of sample or MDA standard, 50 µl of 0.05% butylated hydroxytoluene (BHT), 400 µl of 0.44 M H_3_PO_4_, and 100 µl of 0.42 mM 2-thiobarbituric acid (TBA) were added and heated for 1 h on a 100°C, followed by 250 µl *n*-butanol extraction of the MDA–TBA derivative. The HPLC mobile phase comprised of acetonitrile–buffer (20∶80, v/v, buffer 50 mM KH_2_PO_4_, pH of 6.8 adjusted with KOH). The column was an ESA 150×3-mm C18 column with particle size of 3 µm (ESA, Inc.). The fluorescence detector was set at an excitation wavelength of 515 nm and emission wavelength of 553 nm. The concentration of MDA is expressed as nmol per mg protein.

### GC/MS analysis for F_2_-Isoprostanes

Membrane-esterified F_2_-Isoprostanes (F_2_-IsoPs) were measured by stable isotope dilution, negative-ion chemical ionization gas chromatography/mass spectrometry using a ^4^H_2_-8-iso PGF_2α_ internal standard as previously described [47]. Briefly, brain tissue samples were homogenized in 2∶1 chloroform:methanol containing 0.5% butylated hydroxytolulene and lipids were extracted by the method of Folch [58]. Esterified F_2_-IsoPs were hydrolyzed by chemical saponification with 15% potassium hydroxide, extracted using C_18_ and silica Sep-Pak cartridges (Waters Corporation, Milford, MA), purified by thin-layer chromatography, converted to pentafluorobenzyl ester trimethylsilyl ether derivates, and quantified using gas chromatography/negative ion chemical ionization/mass spectrometry. Quantification of F_2_-IsoPs was performed by comparison to a known amount of ^4^H_2_-8-iso PGF_2α_ internal standard.

### HPLC analysis for 8OH2dG and dG

DNA extraction and HPLC analysis methods were modified from a previous report [59]. Briefly, rat cortex DNA was extracted using TRIzol reagent (Invitrogen, CA, USA) with the inclusion of 1 mM deferoxamine mesylate (DFOM). DNA pellets in 80 ul of H_2_O were hydrolyzed by adding 10 µl of Nuclease P1 (0.4 U/µl in 300 mM sodium acetate, 0.2 mM ZnCl_2_, pH 5.3), and 5 µl of alkaline phosphatase (1 U/µl). The hydrolysate (100 µl) was mixed with 2 µl of 5 M perchloric acid and centrifuged at 18,000 *g* for enzyme removal. The supernatant (50 µl) was isocratically eluted through a 4.6×250 mm C18 column (ESA, Inc Chelmsford, MA) with a mobile phase containing 20 mM LiH_2_PO_4_, 4.0 mM 1-octanesulfonic acid and 1% (v/v) methanol and detected first by a 2-channel Coulochem II electrochemical detector (ESA, Inc. Chelmsford, MA), set with potentials of Channel 1 at 165 mV and Channel 2 at 300 mV for 8OH2dG, and followed by a Waters 486 UV detactor set with a wavelength at 260 nm for dG. DNA oxidation was indicated by the concentration ratio of 8OH2dG ×10^3^ vs. dG.

### HPLC analysis for GSH and GSSG

Brain tissues were homogenized while SH-SY5Y cells were sonicated in chilled 0.1 M perchloric acid and centrifuged. The supernatants were taken for HPLC as modified from a reported method [60]. Breifly, 15 ul supernatant was isocratically eluted through a 4.6×150 mm C18 column (ESA, Inc Chelmsford, MA) with a mobile phase containing 50 mM LiH_2_PO_4_, 1.0 mM 1-octanesulfonic acid and 1.5% (v/v) methanol and detected by a 2-channel Coulochem III electrochemical detector (ESA, Inc. Chelmsford, MA), set with guard cell potential 950 mV, Channel 1 potential 500 mM for GSH detection and Channel 2 potential 880 mV for GSSG detection. Concentrations of GSH and GSSG are expressed as nmol per mg protein.

### Immunohistochemistry and stereologic analysis

Tissues for histological analysis were fixed with 4% paraformaldehyde in 0.1 M phosphate buffer (pH 7.4) and then cryoprotected in 30% sucrose overnight at 4°C. A modified avidin-biotin-peroxidase technique was employed for immunohistochemistry. Briefly, 35 micrometer thick coronal brain sections were pretreated with 3% H_2_O_2_ in 0.1 M PBS for 30 min. The sections were rinsed in PBS twice for 5 min each. The sections were incubated sequentially in (a) 1% bovine serum albumin (BSA)/0.2% Triton X-100 (Sigma, St. Louis, MO) for 30 minutes, (b) appropriate primary antibodies (rabbit anti-TH affinity purified antibody (1∶4,000; Chemicon, Temecula, CA), rabbit immunoaffinity purified anti-nitrotyrosine (4 µg/ml; Upstate Biotechnology, Lake Placid, NY), rabbit polyclonal anti-malondialdehyde (1∶1,000; provided by Dr. C. Thomas, Hoechst Marion Roussel), mouse anti-α-synuclein (1∶1,000; BD Transduction Laboratories, San Jose, CA), mouse anti-NeuN (1∶1,000; Chemicon, Temecula, CA)) (diluted in PBS/0.5% BSA) for 18 hours, (c) appropriate biotinylated IgG (1∶200, diluted in PBS/0.5% BSA, Vector Laboratories, Burlingame, CA) for 1 hour, and (d) avidin-biotin-peroxidase complex (1∶200 in PBS; Vector Laboratories) for 1 hour. The immunoreaction was visualized using 3,3′-diaminobenzidine tetrahydrochloride dihydrate (DAB) with nickel intensification (Vector Laboratories) as the chromogen. All incubations and rinses were performed at room temperature with agitation using an orbital shaker. The sections were mounted onto gelatin-coated slides, dehydrated, cleared in xylene and coverslipped.

For stereological cell counts serial coronal sections (50 µm) were cut through the substantia nigra using a cryostat. Two sets of sections were prepared with each set consisting of 7–8 sections, 100 µm apart. One set of sections was stained with Nissl (cresyl violet). Another set was processed for TH immunohistochemistry as described above. The numbers of Nissl-stained or TH-immunoreactive neurons in the substantia nigra pars compacta were counted using the optical fractionator method in the Stereo Investigator (v 4.35) software program (Microbrightfield, Burlington, VT).

A qualitative examination was performed to analyze immunoreactivities of α-synuclein, malondialdehyde and nitrotyrosine. The analysis was done using 7 sections per animal (n = 5 per group).

One set of serial sections (210 µm apart) immunostained using a mouse antibody against the neuronal marker NeuN were measured for areas of 3-NP-induced NeuN loss to determine the lesion volume using the point-counting method and the cavalieri principle as estimator in the same software program.

### Statistical analysis

Data represent mean±standard error of means (SEM) from groups of animals. Experimental analysis applied with one-way ANOVA. When F values implied significance at a level p<0.05, Student-Newman-Keul's or Tukey–Kramer multiple comparison tests was applied to determine where the differences among groups arose. All statistical analysis was performed using the Graphpad Instat software (GraphPad, San Diego, CA).
